# Altered transcriptome signature of phenotypically normal skin fibroblasts heterozygous for CDKN2A in familial melanoma: relevance to early intervention

**DOI:** 10.18632/oncotarget.786

**Published:** 2013-01-19

**Authors:** Meiyun Fan, Susan R. Pfeffer, Henry T. Lynch, Pamela Cassidy, Sancy Leachman, Lawrence M. Pfeffer, Levy Kopelovich

**Affiliations:** ^1^ Department of Pathology and Laboratory Medicine, and the Center for Cancer Research, University of Tennessee Health Science Center, Memphis, TN, USA; ^2^ Department of Preventive Medicine, Creighton University, Omaha, NE, USA; ^3^ Melanoma & Cutaneous Oncology Program, Huntsman Cancer Institute, and Department of Dermatology, University of Utah, Salt Lake City, UT, USA; ^4^ Division of Cancer Prevention, National Cancer Institute, Bethesda, MD, USA

**Keywords:** Familial Melanoma, gene expression, CDKN2A, p16, Mutations, Melanoma

## Abstract

Familial melanoma (FM) is a dominantly heritable cancer that is associated with mutations in the tumor suppressor CDKN2A/p16. In FM, a single inherited “hit” occurs in every somatic cell, enabling interrogation of cultured normal skin fibroblasts (SFs) from FM gene carriers as surrogates for the cell of tumor origin, namely the melanocyte. We compared the gene expression profile of SFs from FM individuals with two distinct CDKN2A/p16 mutations (V126D-p16 and R87P-p16) with the gene expression profile of SFs from age-matched individuals without p16 mutations and with no family history of melanoma. We show an altered transcriptome signature in normal SFs bearing a single-hit inherited mutation in the CDKN2A/p16 gene, wherein some of these abnormal alterations recapitulate changes observed in the corresponding cancer. Significantly, the extent of the alterations is mutation-site specific with the R87P-p16 mutation being more disruptive than the V126D-p16 mutation. We also examined changes in gene expression after exposure to ultraviolet (UV) radiation to define potential early biomarkers triggered by sun exposure. UV treatment of SFs from FM families induces distinct alterations in genes related to cell cycle regulation and DNA damage responses that are also reported to be dysregulated in melanoma. Importantly, these changes were diametrically opposed to UV-induced changes in SF from normal controls. We posit that changes identified in the transcriptome of SF from FM mutation carriers represent early events critical for melanoma development. As such, they may serve as specific biomarkers of increased risk as well as molecular targets for personalized prevention strategies in high-risk populations.

## INTRODUCTION

Skin cancer is the most common human cancer worldwide [1]. Melanoma is a highly aggressive form of skin cancer that accounts for ~80% of skin cancer-related deaths in the US. Genetic predisposition and UV radiation are the major risk factors for skin cancer, including melanoma [2]. The clustering of melanoma within a single family that is characterized by multiple atypical nevi is referred to as familial melanoma (FM) [3]. FM is likely to occur at an early age, in multiple generations and with high penetrance, resulting in a 35- to 70-fold increase in the risk of developing melanoma [4, 5]. Significantly, a proclivity towards pancreatic cancer is also evident in these families [6-9].

CDKN2A mutations occur in approximately 20-40% of melanoma-prone families world-wide [10]. Variable rates of mutations have been found in sporadic melanomas; in some studies being as high as 50% in primary lesions [11]. The CDKN2A gene locus generates two proteins through alternate slicing: p16^INK4a^ and p14^arf^. The p16^INK4a^ protein binds to CDK4 and CDK6, inhibiting their ability to phosphorylate the retinoblastoma protein. The p14^arf^ protein stabilizes the tumor suppressor protein p53. Collectively, these CDKN2A isoforms are potent tumor suppressors that play distinct but critical roles in cell cycle progression and apoptosis [12]

Although heterozygous loss of p16^INK4a^ function is sufficient to confer a 67% lifetime risk of melanoma [13], the mechanisms responsible for tumor enhancement have yet to be clarified [14, 15]. Mechanistic studies have been hampered by the difficulty of growing in vitro a sufficient quantity of the target cell, the human melanocyte [16].We leveraged the presence of heterozygous CDKN2A mutations in all somatic cells of FM affected individuals to study skin fibroblasts (SFs) as surrogates for melanocytes. We reasoned that like other dominantly heritable cancers, CDKN2A mutations carried in phenotypically normal SFs from FM patients could provide clues to the early molecular events that predispose FM melanocytes to malignant conversion (for review see [17]).

In the present study we studied two FM families each with distinct CDKN2A mutations: V126D-p16 and R87P-p16. We show that gene expression profiles are altered in the phenotypically normal SFs from FM families when compared to SFs from normal controls. Further, we show that UV-irradiation of SFs from FM cohorts results in specific alterations in the expression of genes, which regulate cell cycle and DNA damage response, and that similar alterations are also observed in melanoma lesions.

Our data suggest that expression profiling can identify specific, early biomarkers of increased cancer risk in FM and in sporadic melanomas [13, 18]. Importantly, since the molecular changes that occur in “one-hit” lesions do not include confounding secondary and tertiary tumor effects that accumulate during malignant progression, these biomarkers may also represent valuable drug targets, which can be used to guide development of personalized prevention strategies for both FM and melanomas.

## RESULTS

### Gene expression profiling of phenotypically normal SF cultures from FM mutation carriers and unaffected controls

**Figure 1:**
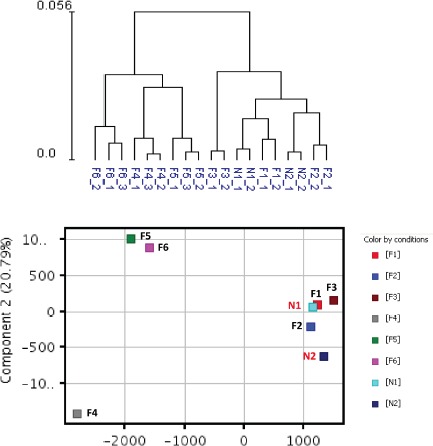
Hierarchical clustering and principal component analysis of gene expression data from SF cultures Unsupervised hierarchical clustering (upper panel) and principal component analysis (lower panel) were performed on replicate cultures representing normal individuals with wild-type CDKN2A (N) or FM individuals with CDKN2A mutations (F) using normalized expression data of all probes.

Whole genome expression profiling was performed on RNA prepared from SF cultures representing three distinct groups (Table 1). Group 1 consisted of two unaffected spouse controls (N1 and N2) from FM families. They expressed wild-type CDKN2A and had no family history of melanoma; Group 2 consisted of three FM mutation carriers with a V126D-p16 mutation (F1, F2, and F3); Group 3 consisted of three FM mutation carriers (F4, F5 and F6) with a R87P-p16 mutation. Unsupervised hierarchical clustering and principal component analysis (PCA) showed that genes expressed in cultured SF from individuals within each of the three groups clustered closely together (Fig. 1). Notably, V126D-p16 heterozygous mutation carriers (Group 2), on average, co-segregated with normal controls (Group 1).

**TABLE 1:** Normal and familial melanoma individuals included in this study

Patient	Gender	Age	CDKN2A/p16 mutation
N1	Male	51	None
N2	Male	51	None
F1	Male	18	V126D
F2	Male	52	V126D
F3	Male	45	V126D
F4	Female	36	R87P
F5	Female	73	R87P
F6	Female	62	R87P
N, normal individual who has no family history of melanoma F, familial melanoma mutation carrier			

**Figure 2:**
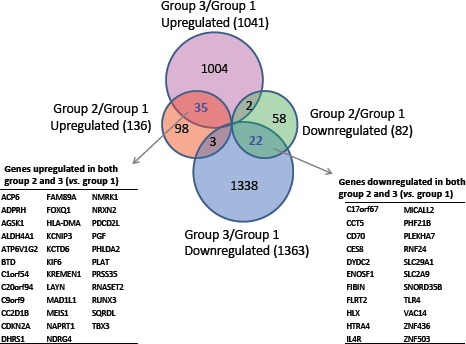
Differentially-expressed genes in familial SFs with distinct CDKN2A mutations The inserted tables in the Venn Diagrams list the genes coordinately upregulated (left panel) or downregulated (right panel) in both Group 2 and Group 3 SFs in comparison to normal SFs (Group1).

Compared to normal controls (Group 1), the expression of 136 and 1041 genes was upregulated (one-side FDR=0.1, fold change≥1.5) in groups 2 and 3, respectively, and the expression of 82 and 1363 genes was downregulated (one-side FDR=0.1, fold change≥1.5) in Groups 2 and 3, respectively. A subset of 35 genes was upregulated in both groups of FM patients (Groups 2 and 3) compared to controls (Group 1), while expression of 22 genes was downregulated in both groups of FM patients compared to Group 1 (Figure 2). The R87P-p16 mutation (Group 3) had a much greater impact on basal gene expression than the V126D-p16 mutation (Group 2). A summary of genes differentially expressed in SF cultures from FM patients is presented in Supplementary Table 1.

**Figure 3:**
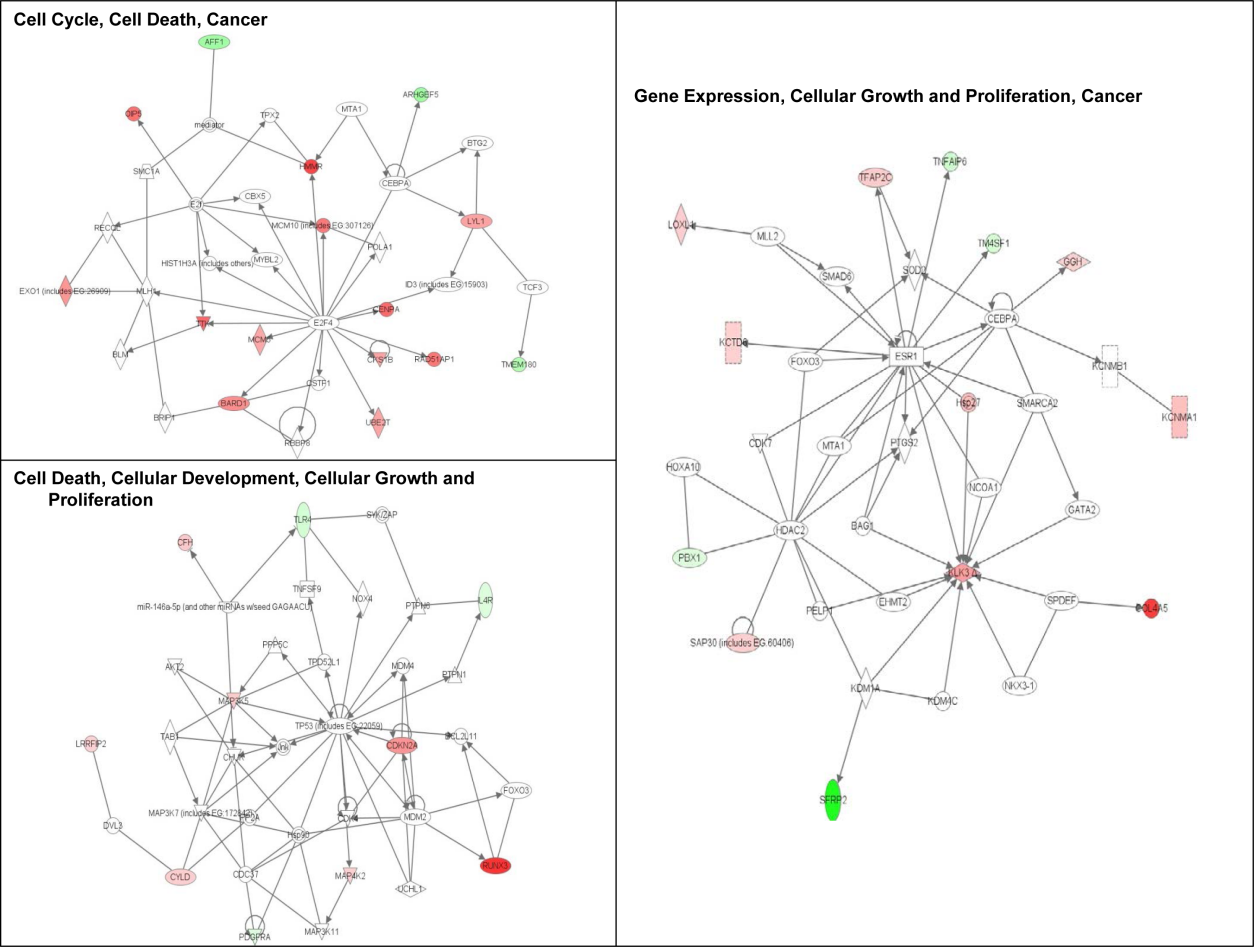
Signaling networks affected by the V126D-p16 mutation Networks of differentially-expressed genes at baseline in familial SFs with the V126D-p16 mutation (Group 2) were generated using Ingenuity Pathway Analysis software. Genes upregulated or downregulated in Group 2 (vs. Group 1) were highlighted in red or green, respectively.

**Figure 4:**
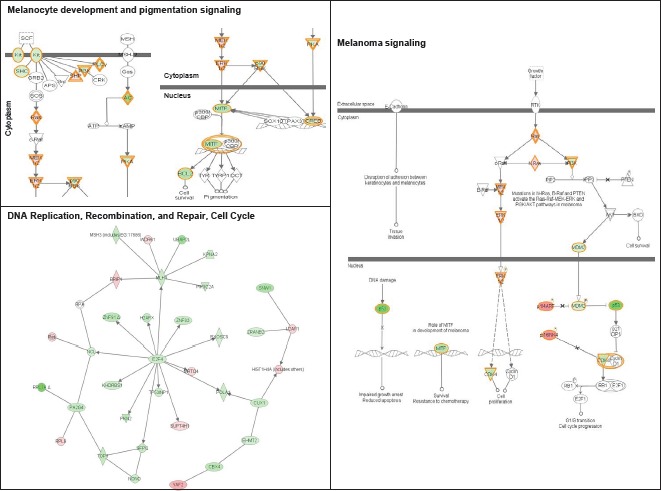
Signaling networks affected by the R87P-p16 mutation Networks of differentially-expressed genes altered at baseline in familial SFs with a R87P-p16 mutation (Group 3) were generated using Ingenuity Pathway Analysis software. Genes upregulated or downregulated in Group 3 (vs. Group 1) were highlighted in red or green, respectively.

Ingenuity Pathway Analysis (IPA) of genes upregulated or downregulated at baseline in Group 2 FM patients showed they were largely associated with cell proliferation, cell death, and cancer (Figure 3). More pathways were differentially affected in Group 3 than in Group 2 and were associated with cell cycle regulation, DNA damage response, DNA repair, and apoptosis. Furthermore, canonical pathways linked to normal melanocyte development and pigmentation, as well as those associated with melanoma signaling, were also affected in the Group 3 FM cohort (Figure 4).

We further examined the overlap between genes differentially expressed at baseline in both groups of SF cultures derived from FM patients and genes differentially expressed in melanoma (Gene Expression Omnibus datasets GDS1375 and GDS1989). A total of seven melanoma-associated genes were found coordinately altered in Group 2 and 3 SF cultures (Supplementary Table 1). Specifically, FLRT2 was downregulated while MAD1L1, PHLDA2, PLAT, CC2D1B, CDKN2A and RUNX3 were upregulated in both melanoma and in Group 2 and Group 3 SFs. Not surprisingly, the number of genes differentially expressed in melanoma is far more extensive than in normal-appearing SFs. The more extensive changes in melanoma likely represent secondary or tertiary changes not directly related to the etiology of melanoma, while the altered gene signatures in normal-appearing SFs from FM cohorts are critical to the initiated state.

### UV sensitivity of normal controls and FM fibroblasts

**Figure 5:**
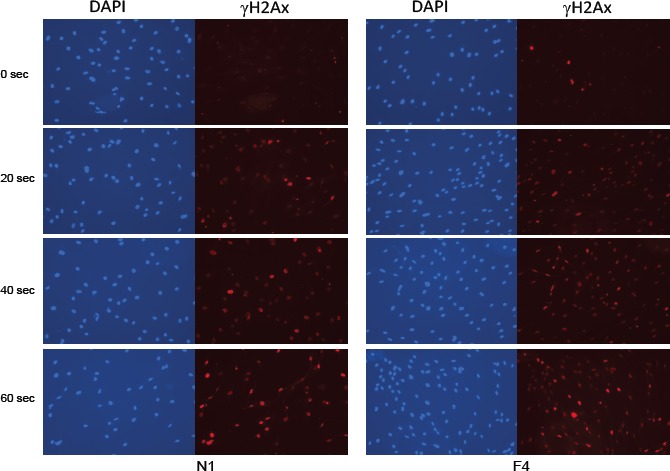
Induction of DNA damage by UV irradiation in SF cultures SFs from a familial individual (F4) and a normal control individual (N1) were UV-irradiated for 20, 40 or 60 seconds, and then fixed in 3.7% formaldehyde at 2 hr after UV exposure. Double-stranded DNA breaks were visualized by γH2Ax immunofluorescence and nuclei were counterstained with DAPI.

Repeated UV exposure can lead to DNA damage and the development of skin cancer. We confirmed that UV induces DNA damage in SF cultures from both FM individuals (Group 2 and 3) and normal controls (Group 1) using γH2Ax immunofluorescence as a measure of double-stranded DNA breaks. As shown in Figure 5, after 40 seconds of UV exposure, 75-80% of the SFs had DNA strand breaks. This is consistent with previous studies showing that SF cultures from individuals with an autosomal dominant colon cancer syndrome are not abnormally sensitive to DNA damaging agents such as UV radiation [19].

### Altered gene expression induced by UV-exposure of FM fibroblasts

**Figure 6:**
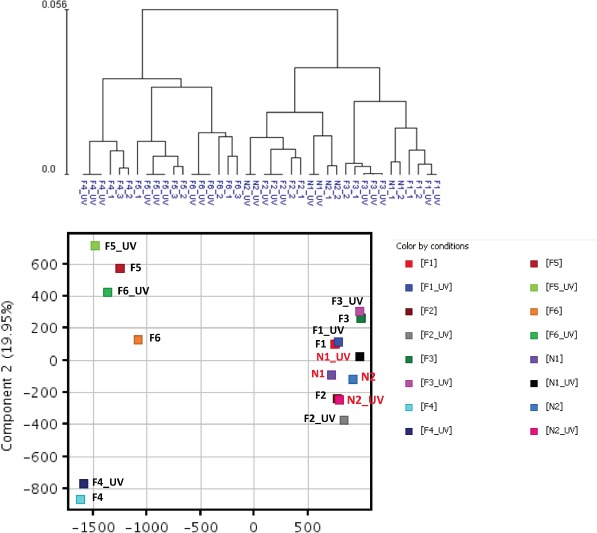
Hierarchical clustering and principal component analysis of gene expression data from SF cultures before and after UV irradiation Unsupervised hierarchical clustering (upper panel) and principal component analysis (lower panel) were performed on gene expression data from un-irradiated and UV-irradiated SF cultures from the three cohorts using normalized expression data of all probes.

**Figure 7:**
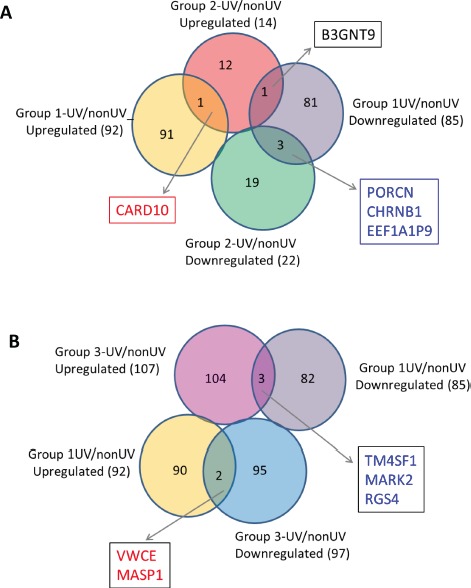
UV-affected genes in normal SFs and familial SFs with distinct CDKN2A mutations Panel A. Venn diagrams of the UV-affected genes in normal SFs (Group 1) and familial SFs with a V126D-p16 mutation (Group 2). Four genes were coordinately regulated by UV irradiation (3 downregulated and 1 upregulated) in the two groups of SF cultures. Panel B. Venn diagrams of the UV-affected genes in normal SFs (Group 1) and familial SFs with a R87P-p16 mutation (Group 3). Five genes were oppositely regulated by UV irradiation in these two groups of SF cultures.

Next we determined the effect of UV exposure on gene expression in SF cultures from normal and FM individuals (Supplementary Table 2). The gene expression distribution clusters among the three groups following UV irradiation (Figure 6) was similar to that seen before UV irradiation (Figure 1). Figure 7 shows pair-wise comparison analysis of UV-irradiated vs. un-irradiated gene expression for each group (one-side FDR=0.1, fold change ≥1.5). UV irradiation of normal SF cultures led to the upregulation of 92 genes and downregulation of 85 genes. Expression of 22 and 97 genes was downregulated by UV in Group 2 and 3, respectively, and expression of 14 and 107 genes was upregulated in Group 2 and 3, respectively. However, expression of only a few genes was coordinately regulated between FM and normal SF cultures. In Groups 1 and 2 only PORCN, CHRNB1 and EEF1A1P9 were coordinately downregulated by UV, while the expression of only one gene (CARD10) was coordinately upregulated by UV (Figure 7A). In contrast, there were no genes coordinately upregulated or downregulated by UV in Groups 1 and 3 (Figure 7B). Moreover, there was no overlap of UV-responsive genes between the two groups of SFs with the different CDKN2A mutations.

**Figure 8:**
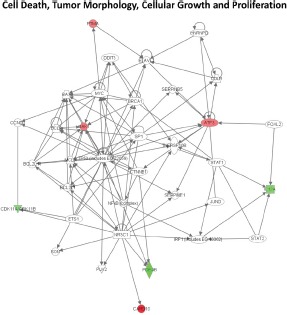
Signaling network of genes affected by UV in familial SFs with a V126D-p16 mutation The gene network was generated using Ingenuity Pathway Analysis software. Genes upregulated or downregulated by UV in Group 2 were highlighted in red or green, respectively.

**Figure 9:**
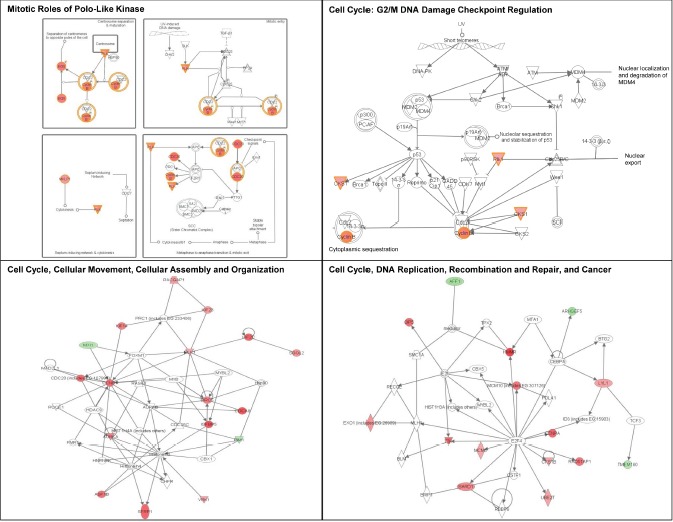
Signaling networks affected by UV in familial SFs with a R87P-p16 mutation The gene networks were generated using Ingenuity Pathway Analysis software. Genes upregulated or downregulated by UV in Group 3 were highlighted in red or green, respectively.

We then performed an IPA analysis of the genes regulated by UV. Genes associated with cell cycle regulation were over-represented in UV-affected genes in both Group 2 and Group 3 SF cultures (Table 2; Figures 8 and 9). As shown in Figure 8, some of the genes specifically altered in Group 2 are associated with cell death, growth and proliferation as well as tumor morphology. Some of the genes specifically altered in Group 3 are associated with regulating the mitotic roles of polo-like kinases and G2/M damage checkpoints as well as with DNA repair and cancer (Figure 9). Interestingly, cell cycle regulatory pathways are overrepresented in both UV-treated Group 2 and 3 cultures, even though the overall gene expression profile at baseline in Group 2 SF is similar to control Group 1. In contrast to FM mutation carriers, and perhaps, most importantly, the expression of genes associated with cell cycle regulation was not altered after UV-irradiation of SF from normal controls (Group 1).

**TABLE 2:** Enriched Ingenuity Function Groups and Signaling Pathways of UV-regulated genes

Enriched Ingenuity Function Groups (Group1-UV vs nonUV)				
Functions Annotation	p-Value		Molecules	# Molecules
cancer	1.69E-02		ADRA1B, AMPH, BTG1, CFB, CGB, CHRNB1, CLEC11A, CLEC3B, COLEC12, CSMD1, DAPK1, DUSP1, ECE2, F8, FCGR2A, FUCA1, GLI2, IFI27, IFT57, IGF2BP3, KCNJ8, KIT, KRT7, LGR4, LMO2, MASP1, MLPH, NDRG1, NPTX1, NR3C1, PHGDH, PRRX1, RASSF4, RGS4, ROBO1, RUNDC3B, SATB1, SEL1L, SEMA3C, SERPING1, SOCS2, STON1, TFAP2A, TGFBR3, TM4SF1, ZFPM2	46
metastasis	2.78E-02		COLEC12, LGR4, MLPH, PRRX1, RGS4, TGFBR3, TM4SF1	7
Enriched Ingenuity Function Groups (Group2-UV vs nonUV)				
Functions Annotation	p-Value		Molecules	# Molecules
proliferation of tumor cell lines	3.87E-02		ATF3, BAK1, CARD10, CDK11A/CDK11B, FLT3LG, PTMA	6
cell cycle progression	9.05E-03		ATF3, CARD10, FLT3LG, IL12A, PTMA	5
Enriched Ingenuity Function Groups (Group3-UV vs nonUV)				
Functions Annotation	p-Value	Activation z-score	Molecules	# Molecules
metastasis	1.31E-04		ANGPTL2, C10orf116, DLGAP5, EPHA2, IGFBP5, MME, MMP3, PAPPA, RGS4, SEL1L3, TM4SF1, TXNIP, VEGFA	13
tumorigenesis	1.37E-08	0.832	ADAMTS5, AFP, ANGPTL2, APOD, ASF1B, AURKA, BARD1, BIRC5, BUB1, C10orf116, CACNA1C, CASD1, CCNB1, CCNB2, CDC20, CDCA8, CDKN3, CENPA, CKS1B, COL7A1, CRIP1, DEPDC1, DLGAP5, DUSP2, ENPP1, EPHA2, EXO1, FAM83D, FBLN5, FUBP1, GBP2, GTF2I, HMMR, HSPB7, IGFBP5, ISOC1, ITGB3, KIF11, KIF14, KIF23, KIF2C, MASP1, MCM10, MCM5, MME, MMP12, MMP3, MXI1, NEK2, OSR2, PAPPA, PAQR4, PDE5A, PDPN, PHLDA2, PIM1, PLK1, PLK4, PLXNC1, PRDM1, PRSS12, PTGER2, RACGAP1, RGS2, RGS4, RNASEH2A, RREB1, SCG2, SCP2, SEL1L3, SFRP1, SLC47A1, SMOX, TM4SF1, TTK, TXNIP, UBE2T, VEGFA, VHL, VRK1, VWA5A, WISP2	82
angiogenesis	6.48E-03	-0.595	ANGPTL2, GTF2I, ITGB3, MMP12, RGS4, RHOJ, SCG2, VEGFA	8
Cell Cycle, arrest in G2 phase	8.56E-03		AURKA, CCNB1, IGFBP5, MXI1, PLK1	5
Cell Cycle, arrest in M phase	3.41E-03		CDC20, NUF2, PLK1	3
Cell Cycle, arrest in mitosis	7.82E-04		BIRC5, BUB1, PLK1, TTK	4
Cell Cycle, cell cycle progression	6.91E-08	-2.71	AURKA, BIRC5, BORA, BUB1, CCNB1, CDC20, CDCA8, CDKN3, CENPA, DLGAP5, IGFBP5, KIF11, KIF2C, MXI1, NDC80, NEK2, NUDT1, NUF2, PIM1, PLK1, SKA1, SKA3, TM4SF1, TTK, VEGFA, VHL	26
Cell Cycle, checkpoint control	1.52E-02		BUB1, CDC20, NDC80	3
Cell Cycle, cleavage of cells	1.89E-02		BIRC5, FUBP1, SKA1	3
Cell Cycle, cytokinesis	1.40E-07	2.157	AURKA, BIRC5, CCNB1, CDC20, KIF14, KIF23, PIM1, PLK1, RACGAP1, TM4SF1	10
Cell Cycle, delay in initiation of M phase	1.96E-05	-0.152	BIRC5, CCNB1, PIM1, PLK1	4
Cell Cycle, G2/M phase	3.34E-03		AURKA, BIRC5, CCNB1, IGFBP5, MXI1, PLK1	6
Cell Cycle, interphase	2.66E-02		AURKA, BIRC5, CCNB1, CDKN3, IGFBP5, MCM10, MXI1, PIM1, PLK1, TXNIP, VHL	11
Cell Cycle, M phase	6.50E-11	1.803	AURKA, BIRC5, CCNB1, CDC20, CDCA8, DLGAP5, KIF14, KIF23, LRRC6, NUF2, PIM1, PLK1, RACGAP1, SKA1, TM4SF1	15
Cell Cycle, mitosis	5.16E-11	-1.769	AURKA, BIRC5, BORA, BUB1, CCNB1, CDC20, CDCA8, CENPA, DLGAP5, IGFBP5, KIF11, KIF2C, NDC80, NEK2, NUF2, PLK1, SKA1, SKA3, TTK, VEGFA	20
Cell Cycle, senescence of cells	4.08E-03	-1.467	BUB1, CENPA, NUDT1, TM4SF1, VEGFA	5
Cell Cycle, spindle checkpoint of cells	1.33E-06		BIRC5, BUB1, DLGAP5, PLK1, TTK	5
cell death	1.98E-02	-0.295	AFP, AURKA, BARD1, BIRC5, CCNB1, CDC20, DEPDC1, EPHA2, FUBP1, HMMR, IGFBP5, IL17D, ITGB3, ITGB3BP, KIF11, KIF14, MCM10, MME, NDC80, NDP, NEK2, NFKBIZ, NUAK2, NUF2, PHLDA2, PIM1, PLK1, PRDM1, PTPN13, RGS4, RIPK3, SCG2, SFRP1, SLC47A1, SMOX, TTK, VEGFA, VHL	38
differentiation of cells	5.88E-03	-0.678	ANGPTL2, BHLHE41, ENPP1, IGFBP5, IL17D, ITGB3, PDE5A, PRDM1, PRKX, RACGAP1, RGS4, SFRP1, TXNIP, VEGFA, VHL	15
proliferation of cells	5.81E-05	1.245	AFF1, AFP, APOD, AURKA, BARD1, BIRC5, BUB1, CDCA8, CDKN3, CKS1B, CRIP1, DLGAP5, ENPP1, EPHA2, ERBB2IP, FABP3, HMMR, IGFBP5, ITGB3, KCNN4, KIF11, KIF2C, LEPREL1, MCM5, MMP12, MXI1, NDP, NEK2, NFATC4, NUDT1, PDE5A, PENK, PHLDA2, PIM1, PLK1, PRDM1, PTGER2, PTPN13, RACGAP1, RGS4, SCG2, SFRP1, SMOX, STEAP2, TTK, TXNIP, UTP20, VEGFA, VHL, WISP2	50
invasion of cells	7.58E-03	-0.187	AURKA, EPHA2, FBLN5, HMMR, ITGB3, MMP3, NUAK2, PAPPA, PTGER2, RGS4, SFRP1, VEGFA, VHL	13
DNA damage	9.86E-03	-1.922	BIRC5, MCM10, PLK1, SMOX	4
Enriched Ingenuity Canonical pathways (Group3-UV vs nonUV)				
Ingenuity Canonical Pathways	-log(p-value)	Ratio	Molecules	
Mitotic Roles of Polo-Like Kinase	5.22E+00	1.09E-01	KIF23, PLK4, CDC20, CCNB2, PLK1, KIF11, CCNB1	
Cell Cycle: G2/M DNA Damage Checkpoint Regulation	2.86E+00	8.33E-02	CKS1B, CCNB2, PLK1, CCNB1	
HIF1A Signaling	1.57E+00	3.92E-02	VEGFA, MMP3, MMP12, VHL	
cAMP-mediated signaling	1.48E+00	2.82E-02	RGS2, NPR3, ADCY4, RGS4, PDE5A, PTGER2	
Enriched Ingenuity Function Groups (Overlap_Group3-UV vs nonUV and Melanoma vs normal skin)				
Functions Annotation	p-Value	Activation z-score	Molecules	# Molecules
proliferation of cells	8.79E-04	1.247	AFF1, APOD, AURKA, BIRC5, BUB1, CDKN3, DLGAP5, ENPP1, HMMR, KIF2C, PHLDA2, PIM1, PLK1, PTPN13, SCG2, TTK, TXNIP	17
cell death	2.04E-04	-1.034	AURKA, BIRC5, CCNB1, CDC20, DEPDC1, HMMR, IL17D, KIF14, NDC80, NUF2, PHLDA2, PIM1, PLK1, PTPN13, SCG2, TTK	16
cell cycle progression	6.97E-08	-1.916	AURKA, BIRC5, BUB1, CCNB1, CDC20, CDKN3, DLGAP5, KIF2C, NDC80, NUF2, PIM1, PLK1, TTK	13
Enriched Ingenuity Canonical Groups (Overlap_Group3-UV vs nonUV and Melanoma vs normal skin)				
Ingenuity Canonical Pathways	-log(p-value)	Ratio	Molecules	
Mitotic Roles of Polo-Like Kinase	4.57E+00	6.25E-02	CDC20, CCNB2, PLK1, CCNB1	
Cell Cycle: G2/M DNA Damage Checkpoint Regulation	3.56E+00	6.25E-02	CCNB2, PLK1, CCNB1	

**Figure 10:**
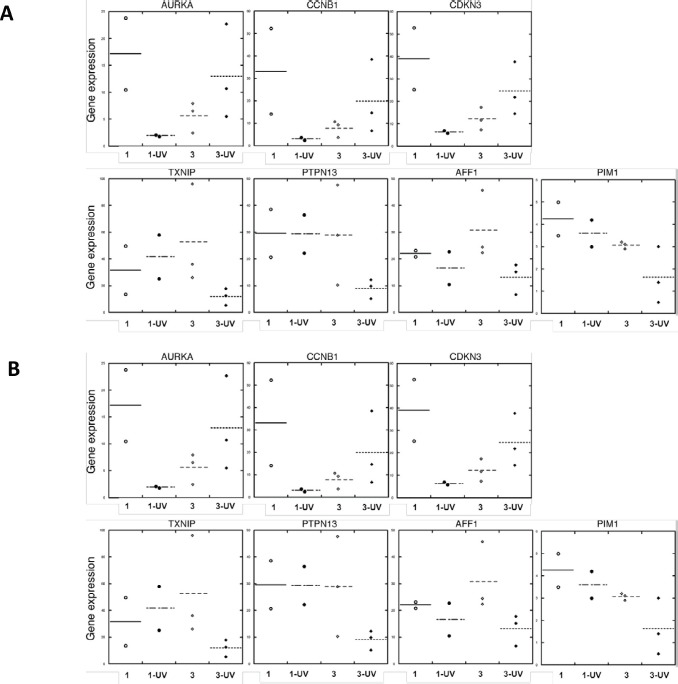
Expression in normal skin and melanoma tissue of genes affected by UV in familial SFs with a R87P-p16 mutation RNA was isolated from (Panel A) unirradiated and UV-irradiated SFs from Group 1 (normal) and Group 3 (a R87P-p16 mutation), or (Panel B) normal skin and melanoma tissue, and mRNA levels of the indicated genes were measured by qPCR, normalized to β-actin expression, and presented as means. The horizontal line shows the mean value for all members of each group of samples.

To further determine the biological significance of our findings, we compared UV-treated, differentially expressed gene sets in FM fibroblasts with published datasets that account for melanoma development. Thus, genes upregulated in melanoma (Omnibus datasets GDS1375 and GDS1989), were also found to be enriched in UV-upregulated genes of Group 3 SF cultures (Fisher's exact one-tailed P value = 0.0158). Similarly, genes downregulated in melanoma were found to be enriched in UV-downregulated genes of Group 3 SF cultures (Fisher's exact one-tailed P value = 0.0043). These differences were not seen in the case of SF from group 2 vs. melanoma. An IPA analysis showed that genes coordinately regulated by UV in Group 3 (UV vs. untreated) SF cultures and in melanoma (vs. normal skin) are involved in cell cycle regulation and apoptosis (Table 2). We therefore validated that the gene expression of AURKA, CCNB1 and CDKN3 was upregulated upon UV-irradiation of Group 3 FM SF cultures (Figure 10A) and compared the expression of these genes in normal skin and melanoma tissue (Figure 10B). As shown in Figure 10B the expression of AURKA, CCNB1 and CDKN3 was enhanced in melanoma tissue as compared to their expression in normal donor skin. In addition, the expression of AFF1, PIM1, PTPN13 and TXNIP was downregulated in UV-irradiated FM SFs with a R87P-CDKN2A mutation (Figure 10A), and the expression of these genes was also found to be downregulated in melanoma tissue (Figure 10B).

## DISCUSSION

Alterations in gene expression associated with single-hit mutations of tumor suppressor genes in phenotypically normal cells appear to represent the earliest molecular changes during cancer development. Previous studies showed that the altered transcriptome and proteome signatures of phenotypically normal cells bearing these single inherited hits parallel many changes observed in the corresponding form of cancer (for review see [17]). Thus, identification of these early alterations may help identify critical molecular targets in both the inherited and sporadic cancers.

In the present study we compared global gene expression profiles of normal SF from control individuals and phenotypically normal skin-derived fibroblasts from FM individuals with two different CDKN2A/p16 mutations. We examined differences in gene expression profile at baseline and following UV irradiation, leading to the following important findings.

First, the transcriptome signatures are altered by single hit CDK2NA/p16 mutations in phenotypically normal SF from FM families. The extent of altered gene expression is mutation-site specific, with R87P-p16-CDKN2A mutation being far more disruptive than V126D-p16 mutations. This is in agreement with previous studies showing that the R87P-p16 mutation appears to have a more severe phenotype in vitro. The R87P-p16 mutation is an inactivating mutation of CDK4/6 binding [15], inhibits kinase activity [20], results in the accumulation of G1 phase cells [20], and has a predominantly nuclear localization [21]. In contrast, results on the V126D-p16 mutation have been less clear, which may reflect its temperature-sensitive biological effects in vitro and ability to partially “retain” wild type CDK4/6 inhibition [15].

Second, the differentially expressed genes at baseline in R87P-p16 mutation carriers exhibit changes in canonical signaling pathways associated with normal melanocyte development and melanomas. KIT and its downstream effector MITF are part of the canonical signaling pathway involved in melanocyte development and pigmentation. KIT signals via MAPK to activate MITF, which in turn is a potent transcriptional and lineage specific regulator of the three major melanocyte pigment enzymes: tyrosinase, TRP1/TyrpI and TRP2/Dct [22].

A third important finding is that UV-irradiated SF from FM patients show deregulation of cell cycle and DNA damage response pathways that are diametrically opposite to the gene expression pattern seen following irradiation of fibroblasts from normal controls. For example, cell cycle related genes were downregulated in UV-irradiated fibroblasts from normal individuals, but these genes were upregulated in fibroblasts derived from FM individuals. Moreover, many of the changes in gene expression seen after UV-irradiation of fibroblasts derived from FM individuals are shared with a previously described Gene Expression Omnibus data set of genes deregulated in malignant melanomas, but not in benign nevi [14, 23]. For example, the protein tyrosine phosphatase PTPN13 has been proposed to function as a tumor suppressor; this is in line with its downregulation [24]. In addition, AFF1 is coordinately downregulated in UV-irradiated fibroblasts and in melanoma, and is often dysregulated in cancer [25].

A fourth important observation is that occurrence of the more “benign” V126D-p16 mutation or of the more “disruptive” R87P-p16 mutation within a given FM family is mutually exclusive, supporting the notion that personalized, cohort-based, intervention modalities, including clinical outcome should be adjusted accordingly. We posit that, direct or up/down-stream “pharmacologic normalization” of the R87P-p16 mutation, individually and together with BRAF intervention [26], may enable more effective strategies to delay or even prevent FM, including the occurrence of melanoma in individuals who are otherwise at risk. In this regard, proclivity towards pancreatic cancer that is likely to be caused by CDKN2A mutations [6-8], particularly among affected carriers of the R87P-p16 mutation, should be explored for early diagnosis/prognosis and intervention of pancreatic cancer.

Together, these data suggest that genomic profiling of phenotypically normal SF can help identify novel molecular targets for chemoprevention, including early specific biomarkers of melanoma risk among individuals who are heterozygous for CDKN2A mutations within FM families; these potential biomarkers would enable mechanism-based early detection of melanoma and personalized prevention strategies to target sporadic melanoma.

## METHODS

### Cell culture

Human SFs derived from normal and FM individuals (Table 1) were cultured in DMEM with 10% Fetal Calf Serum (FCS). Cells were maintained in the presence of penicillin (100 IU/ml), streptomycin (100 mg/ml) and fungizone (250 ng/ml) at 37°C with 5% CO_2_. Medium was changed every 2-3 days, and cells were passaged at confluence at 1:3.

### Microarray Analysis

Total RNA from three independent experiments was prepared using miRNeasy Mini kit (QIAGEN, Valencia, CA) and submitted to the UTHSC Center of Genomics and Bioinformatics (Memphis, TN) for labeling and hybridization to HT-12 expression BeadChips (Illumina Inc.). RNA integrity was validated on an Agilent bioanalyzer, and all samples showed distinct peaks corresponding to intact 28S and 18S ribosomal RNA. Hybridization signals were processed using Illumina GenomeStudio software (annotation, background subtraction, Quantile normalization and presence call filtering). The processed data were filtered to exclude probes with detection p value >0.05 in more than half of the samples. In addition, the hybridization signal intensities <40 were set to the flooring value of 40. GeneSpring GX software (Agilent Technologies) and Expander (Expression Analyzer and Displayer with the R environment) were used for statistical computing and graphics [27]. SAM (Significance Analysis of Microarray) was used to detect probes that demonstrate differential expression among the different subsets of samples (Group 1, 2 and 3_untreated, n=3; Group 1, 2 and 3_UV irradiated, n=3; each subset contains 2-3 biological duplicates) with one-side FDR=0.1. The differentially expressed genes were subjected to fold change analysis. UV-regulated genes were defined as genes with FC≥1.5 (UV treated vs. untreated) and were further subjected to functional annotation and signaling pathway mapping using the Ingenuity Pathway Analysis software (IPA, Ingenuity Systems, Inc.) [28], and clinical relevance analysis using the expression array data of melanoma tissues deposited in Gene Expression Omnibus (GDS1375 and GDS1989).

### Quantitative Real time-PCR

Quantitative real time-PCR (qPCR) was performed using gene-specific primers (sequences in Table S2) on the iCyclerIQ detection system (BioRad, Hercules, CA) using iScript One-Step RT-PCR Kit with SYBR Green (BioRad) as previously described [29, 30]. Reaction parameters were as follows: cDNA synthesis at 50°C for 20 min, iScript reverse transcriptase inactivation at 95°C for 5 min, PCR cycling at 95°C for 10 sec and 60°C for 30 sec for 40 cycles. Gene expression data was normalized to the expression of the β-actin housekeeping gene. The relative units were calculated from a standard curve, plotting 3 different concentrations against the PCR cycle number at the cycle threshold (with a 10-fold increment equivalent to ~3.1 cycles).

### UV-irradiation

Human SFs were grown on glass coverslips in 6-well plates in DMEM with 20% FCS and Penicillin/Streptomycin. Before irradiation the media was aspirated and cells were washed twice with PBS. The coverslips were transferred to a Petri dish containing PBS. The dishes were placed in the UV chamber of a BioRad GS Gene Linker and irradiated with UV light at 2 mJoules/cm^2^. Following irradiation the coverslips were transferred to new 6-well plates, fresh media was added and cells were returned to the incubator. Induction of DNA damage by UV irradiation was examined using γH2Ax immunofluorescence as a measure of double-stranded DNA breaks.

## Supplementary Tables


